# Hypersensitivity reactions with first-line antituberculosis drugs and outcomes of rapid desensitizations

**DOI:** 10.1016/j.waojou.2023.100862

**Published:** 2024-01-03

**Authors:** Gozde Koycu Buhari, Ferda Oner Erkekol, Ilkay Koca Kalkan, Hale Ates, Gurgun Tugce Vural Solak, Ozgur Akkale, Kurtulus Aksu

**Affiliations:** a University of Health Sciences, Ankara Ataturk Sanatoryum Training and Research Hospital, Department of Immunology and Allergy, Ankara, Turkey; b Medicana International Ankara Hospital, Clinic of Immunology and Allergy, Ankara, Turkey; c Etlik City Hospital, Department of Immunology and Allergy, Ankara, Turkey

**Keywords:** Tuberculosis, Antituberculosis, Drug hypersensitivity, Desensitization, Breakthrough reaction

## Abstract

**Background:**

Data about drug hypersensitivity reactions with first-line antituberculosis drugs and their management is limited. Rapid drug desensitization seems to be an appropriate management.

**Objective:**

Evaluate the efficacy of the rapid desensitization protocols in patients who had a reaction phenotypically compatible with immediate-type drug hypersensitivity with first-line antituberculosis drugs and identify possible risk factors of breakthrough reactions during the protocols.

**Methods:**

This is a retrospective study of active tuberculosis patients who had a reaction phenotypically compatible with immediate-type drug hypersensitivity with first-line antituberculosis drugs and underwent desensitization with the drugs used during the reaction. Characteristics of drug hypersensitivity and breakthrough reactions, and outcomes of rapid desensitizations were recorded.

**Results:**

One hundred and seventy-nine patients were included in the study. Most of the initial reactions (n = 132, 73.7%) occurred within the first week of treatment and were mild (n = 146, 81.6%). A total of 690 desensitizations were performed. Desensitizations were successfully completed without any breakthrough reaction in 103 (57.5%) patients and in 29 of 36 (80.6%) patients after a breakthrough reaction. The overall success of desensitizations were found to be 95% (132 of 139 patients). Most of the breakthrough reactions (84%) were mild. Sixteen patients had breakthrough reactions with multiple drugs. Although pyrazinamide was the most common culprit of breakthrough reactions and had the lowest desensitization success, it had the highest rate of a single breakthrough reaction (p < 0.001). Timing of the initial reaction and concomitant breakthrough reaction with ethambutol were found to have increased the risk for breakthrough reaction caused by rifampicin (p = 0.017 and p = 0.010 respectively).

**Conclusion:**

The rapid desensitization protocols used in this study provide a successful and effective management of the patients with a reaction phenotypically compatible with immediate-type drug hypersensitivity with first-line antituberculosis drugs.

Tuberculosis is a serious public health threat unless it is treated properly. Until the SARS-CoV-2 (COVID-19) pandemic, it was the leading cause of death from a single infectious agent, ranking above HIV/AIDS.^1^ The main principle of antituberculosis treatment is to use the safest and most effective drug combination regularly for an adequate time. Treatment of drug-susceptible tuberculosis disease requires combination therapy with first-line antituberculosis drugs, including isoniazid (H), rifampicin (R), pyrazinamide (Z), and ethambutol (E). All 4 drugs are used in the initiation phase of treatment for 2 months, and then H and R are used in the maintenance phase of treatment for 4 months.^2^

Adverse drug reactions and drug hypersensitivity reactions (DHRs) are one of the major causes of noncompliance with antituberculosis treatment.^3^, ^4^, ^5^, ^6^, ^7^, ^8^ Drug provocation testing is the gold standard method to confirm or rule out a DHR and identify the culprit drug.^9^, ^10^, ^11^ However, in the management of antituberculosis DHR, even if the culprit first-line antituberculosis drug is detected, it is not easy to remove it from therapy because there is a limited number of alternative drugs, and second-line antituberculosis drugs are less effective, more expensive, and have more side effects than first-line antituberculosis drugs, and also switching to second-line therapy prolongs the duration of therapy.^1^^,^^12^ It is also known that simultaneous multiple drug hypersensitivity can develop with antituberculosis drugs.^13^, ^14^, ^15^, ^16^, ^17^, ^18^ Detection of the culprit drugs with drug provocation testing will take a long time in such cases. Rapid and appropriate management of patients with antituberculosis drug hypersensitivity is important because prolonged hospitalization and failure of using regular or standard drug combinations are identified as risk factors for drug resistance.^19^^,^^20^ In the management of antituberculosis DHR there are some suggestions about desensitization with all drugs used during hypersensitivity reaction rather than detecting the culprit drug.^21^^,^^22^

We previously defined 6–8 step readministration protocols in patients who had immediate-type DHR to first-line antituberculosis drugs within 1 h after drug intake. Eleven of the 13 patients tolerated the drugs without any breakthrough reaction (BTR). In the remaining 2 patients who had BTRs during protocols, drug tolerance was achieved by modifying the protocol.^23^

The aim of this study is to evaluate the efficacy and safety of the previously defined protocols in a larger patient population who were phenotypically compatible with immediate type DHR with first-line antituberculosis drugs regardless of having a reaction within 1 h and also identify possible risk factors of BTRs during the protocols.

## Materials and methods

### Study population

This is a retrospective observational study of active tuberculosis patients who were phenotypically compatible with immediate-type DHR with first-line antituberculosis drugs and underwent desensitization with the drugs used during the DHR from January 2010 to December 2022 in our Allergy and Clinical Immunology Clinics. The study was approved by the local ethics committee (approval number 2012-KAEK-15/2614).

Inclusion criterias of patients were^1^ phenotypically compatible with immediate-type DHR with first-line antituberculosis drugs and^2^ chronologically developed a reaction within 12 h after the last dose and^3^ desensitized to first-line antituberculosis drug used during DHR. Exclusion criterias of patients were^1^ phenotypically compatible with delayed-type DHR and^2^ insufficient medical records.

In this study, phenotypic immediate-type DHR is defined as a reaction with signs and symptoms such as cutaneous symptoms (pruritus, flushing, urticaria, angioedema), rhinitis, conjunctivitis, respiratory symptoms (dyspnea, bronchospasm), gastrointestinal symptoms (abdominal pain, nausea, vomiting, diarrhea), decrease in blood pressure or anaphylaxis irrespective of chronology. Patients who had a reaction with these signs and symptoms for up to 12 h were included in the study.

Phenotypic delayed-type DHR is defined as a reaction with signs and symptoms such as maculopapular eruption, fixed drug eruption, drug reaction with eosinophilia and systemic symptoms, acute generalized exanthematous pustulosis, symmetrical drug-related intertriginous and flexural exanthemas, Stevens-Johnson syndrome, toxic epidermal necrolysis, serum sickness-like reactions, organ specific delayed drug reactions or vasculitis which were exclusion criteria of the study.

### Patients, disease, DHR, and BTR characteristics

Baseline data including patients’ demographics (age, gender, other drug allergies), disease characteristics (site of infection, diagnostic method, case definition), DHR characteristics (culprit drugs, interval between antituberculosis treatment initiation and initial DHR, interval between last administered drug dose and DHR, the symptoms and severity of DHR, any treatment interruption before DHR), BTR characteristics (culprit drug, interval between the last applied step and BTR, the symptoms and severity of BTR), and also continuation to the protocols after BTR and protocol success were recorded as documented in hospital medical records.

The site of tuberculosis infection was classified as pulmonary only, extrapulmonary only, and combined pulmonary and extrapulmonary. The diagnostic method was classified as bacteriologically confirmed if a biological specimen is positive by smear microscopy, culture, or real-time polymerase chain reaction; histologically diagnosed if the tissue biopsy sample is suggestive of caseification; clinically diagnosed if the clinical and radiological findings are suggestive of tuberculosis infection. Case definition was classified as new cases who have never been treated for tuberculosis or have taken antituberculosis drugs for less than 1 month; previously treated cases who have received 1 month or more of antituberculosis drugs.

The severity of DHRs and BTRs were graded according to Ring-Messmer classification as Grade 1: cutaneous symptoms (itch, flushing, urticaria, angioedema); Grade 2: Grade 1 plus any of the following nausea, abdominal cramps, rhinorrhea, hoarseness, dyspnea, tachycardia, hypotension, arrhythmia; Grade 3: Grade 2 plus any of the following vomiting, defecation, laryngeal edema, bronchospasm, cyanosis, shock; and Grade 4: Grade 3 plus respiratory or circulatory arrest.^24^

### Desensitization protocols

Desensitization was performed with the protocols we described in our previous study.^23^ Protocols were started after all symptoms of the DHR resolved and an appropriate washout period of drugs used in the treatment of DHR. No premedication was used before the procedure. Written informed consent was obtained from patients before each desensitization procedure.

The drugs used during DHR were administered using 6–8 step desensitization protocols on consecutive days, as described in Table 1. The regimens were initiated with 7.5, 10, 10, and 5 mg of isoniazid, rifampicin, pyrazinamide, and ethambutol respectively. Drug doses were given in each regimen at a maximum 2.5-fold increase every 30 min. Only the interval between the next to last dose and the last dose was 60 min. All regimens were implemented to ensure tolerance to the therapeutic dosage of each drug before advancing to the next drug on the consecutive day. Each day, before starting a new drug application, the therapeutic dose for the drug or drugs that were administered on previous days was given to the patient. Then, the regimen for the application of the next drug was launched. The procedure was performed under close observation with one-on-one nurse-to-patient care. In case of a BTR, drug administration was suspended, and the reaction was treated according to international guidelines.^9^^,^^25^ After complete resolution of the symptoms, the protocol was continued starting from the last tolerated dose. The effectiveness and safety data of desensitization protocol were recorded.

**Table 1:** Rapid desensitization protocols used in the study.

Day 1			Day 2		
Time	Drug doses		Time	Drug doses	
			08:00	Isoniazid 300 mg	
	**Isoniazid**			**Rifampicin**	
09:30 10:00 10:30 11:00 11:30 12:30	Solution A^a^- 1 ml Solution A - 2 ml Solution A - 3 ml 1/8 tablet isoniazid 1/4 tablet isoniazid 1/2 tablet isoniazid	7.5 mg 15 mg 22.5 mg 37.5 mg 75 mg 150 mg	09:30 10:00 10:30 11:00 11:30 12:30	Solution C^c^- 1 ml Solution C- 2 ml Solution C- 5 ml Solution B^b^- 5 ml Solution B- 10 ml Solution B- 11 ml	10 mg 20 mg 50 mg 100 mg 200 mg 220 mg
**Day 3**			**Day 4**		
**Time**	**Drug doses**		**Time**	**Drug doses**	
08:00	Isoniazid 300 mg + Rifampicin 600 mg		08:00	Isoniazid 300 mg + Rifampicin 600 mg+ Pyrazinamide 2000 mg	
	**Pyrazinamide**			**Ethambutol**	
09:30 10:00 10:30 11:00 11:30 12:00 12:30 13:30	Solution E^e^- 2 ml Solution E− 4 ml Solution E− 8 ml Solution E− 16 ml Solution D^d^- 3 ml Solution D- 4 ml 1 tablet pyrazinamide 2 tablet pyrazinamide	10 mg 20 mg 40 mg 80 mg 150 mg 200 mg 500 mg 1000 mg	09:30 10:00 10:30 11:00 11:30 12:00 12:30 13:30	Solution G^g^- 1 ml Solution G- 2 ml Solution G- 4 ml Solution G- 8 ml Solution F^f^- 2 ml Solution F- 4 ml 1 tablet ethambutol 1 + 1/4 tablet ethambutol	5 mg 10 mg 20 mg 40 mg 100 mg 200 mg 500 mg 625 mg
**Day 5**					
**Time**	**Drug doses**				
08:00	Isoniazid 300 mg +Rifampicin 600 mg +Pyrazinamide 2000 mg +Ethambutol 1500 mg				

a Solution A: Suspension was prepared by crushing one tablet of isoniazid (300 mg) into a volume of 40 ml 0.9% NaCl to a final concentration of 7.5 mg/ml.

b Solution B: Suspension was prepared by mixing the content of one capsule of rifampicin (600 mg) into a volume of 30 ml 0.9% NaCl to a final concentration of 20 mg/ml.

c Solution C: Suspension was made by 1:2 dilution of solution B to a final concentration of 10 mg/ml.

d Solution D: Suspension was prepared by crushing one tablet of pyrazinamide (500 mg) into a volume of 10 ml 0.9% NaCl to a final concentration of 50 mg/ml.

e Solution E: Suspension was made by 1:10 dilution of solution D to a final concentration of 5 mg/ml.

f Solution F: Suspension was prepared by crushing one tablet of ethambutol (500 mg) into a volume of 10 ml 0.9% NaCl to a final concentration of 50 mg/ml.

g Solution G: Suspension was made by 1:10 dilution of solution F to a final concentration of 5 mg/ml

### Statistical analysis

All statistical analyses were performed using the SPSS (statistical package of social sciences) for Windows 16.0 software package. In the evaluation of the data, mean and standard deviation was used for normally distributed data, and median and interquartile range was used for data that did not show a normal distribution. Values and percentages for ratios were determined by descriptive statistical method. In univariate analyses, Chi-square, Fischer, Student's t-test, and Mann- Whitney U tests were used, as appropriate. Multivariate analysis was conducted through a binary logistic regression model and the variables were selected by backward selection with the elimination of variables at a p-value over 0.20. Results was evaluated at a 95% confidence interval. All p values < 0.05 were considered to be statistically significant.

## Results

### Demographics and disease characteristics

A total of 179 patients; 124 (69.3%) female and 55 (30.7%) male with mean age 49.34 ± 16.38 (range: 18–87) were included in the study. Other drug allergies were present in 19 (10.6%) patients.

The diagnosis was pulmonary tuberculosis in 86 (48%), nonpulmonary tuberculosis in 80 (44,7%), and combined pulmonary and nonpulmonary tuberculosis in 13 (7.3%) patients. Tubercular lymphadenitis (n = 48, 26.8%) was the most common type among extrapulmonary tuberculosis, followed by mastitis (n = 11, 6.1%), pleuritis (n = 9, 5%), peritonitis (n = 5, 2.8%), and osteomyelitis (n = 5, 2.8%). Other types of nonpulmonary tuberculosis according to involved organs were as follows; cutaneous (n = 4, 2.2%), genitourinary (n = 3, 1.7%), miliary (n = 3, 1.7%), and nasal, parotid, uvea, pituiatary gland, and hepatic tuberculosis (n = 1, 0.6% each).

The diagnosis was bacteriologically confirmed in 84 (46.9%) patients. Eighty-one (45.3%) patients were diagnosed histologically, and 14 (7.8%) patients were diagnosed clinically. Cases definitions were as new cases in 160 (89.4%) and previously treated cases in 19 (10.6%) patients (See Table 2).

**Table 2:** Demographics, disease and DHR characteristics of patients.

Variables	All patients (n = 179)^a^	BTR positive patients (n = 76)^a^	BTR negative patients (n = 103)^a^	p^b^	Statistical method used
Age (mean + SD)	49.34 ± 16.38	48.63 ± 15.43	49.85 ± 17.11	0.623	c
Sex, n (%)				0.832	d
Female	124 (69.3)	52 (68.4)	72 (69.9)		
Male	55 (30.7)	24 (31.6)	31 (30.1)		
Other drug allergy, n(%)	19 (10.6)	10 (13.2)	9 (8.7)	0.343	d
Site of infection, n (%)				0.746	d
Pulmonary only	86 (48.0)	34 (44.7)	52 (50.5)		
Nonpulmonary only	80 (44.7)	36 (47.4)	44 (42.7)		
Combined pulmonary and nonpulmonary	13 (3.7)	6 (7.9)	7 (6.8)		
Case definition, n(%)				0.600	d
New case	160 (89.4)	69 (90.8)	91 (88.3)		
Previously treated case	19 (10.6)	7 (9.2)	12 (11.7)		
Diagnostic method, n(%)				0.502	d
Bacteriological	84 (46.9)	34 (44.7)	50 (48.5)		
Histological	81 (45.3)	34 (44.7)	47 (45.6)		
Clinical	14 (7.8)	8 (10.5)	6 (5.8)		
Interval between last drug administration and DHR, n (%)				0.156	d
<1 h	83 (46.4)	40 (52.6)	43 (41.7)		
1–6 h	70 (39.1)	29 (38.2)	41 (39.8)		
6–12 h	26 (14.5)	7 (9.2)	19 (18.4)		
Treatment interruption before DHR, n (%)	10 (5.6)	5 (6.6)	5 (4.9)	0.745	e
Regimen used during DHR, n (%)				0,730	d
Standard HRZE combination	169 (91.6)	69 (90.8)	95 (92.2)		
Non-standard combination	15 (8.4)	7 (9.2)	8 (7.8)		
Initial DHR severity, n (%)				0.226	d
Grade 1	146 (81.6)	58 (76.3)	88 (85.4)		
Grade 2	22 (12.3)	11 (14.5)	11 (10.7)		
Grade 3	11 (6.1)	7 (9.2)	4 (3.9)		
Initial DHR clinical symptoms, n (%)					e
Pruritus with erythema	178 (99.4)	76 (100.0)	102 (99.0)	1000	
Urticaria	100 (55.9)	41 (53.9)	59 (57.3)	0.657	
Angioedema	25 (14.0)	12 (15.8)	13 (12.6)	0.546	
Nausea	19 (10.6)	10 (13.2)	9 (8.7)	0.343	
Vomiting	7 (3.9)	5 (6.6)	2 (1.9)	0.136	
Dyspnea	7 (3.9)	4 (5.3)	3 (2.9)	0.460	
Conjunctivitis	6 (3.4)	3 (3.9)	3 (3.9)	0.700	
Hypotension	6 (3.4)	2 (2.6)	4 (3.9)	1000	
Near-syncope	5 (2.8)	2 (2.6)	3 (2.9)	1000	
Confusion	3 (1.7)	1 (1.3)	2 (1.9)	1000	
Chills	3 (1.7)	2 (2.6)	1 (1.0)	0.575	
Fever	2 (1.1)	1 (1.3)	1 (1.0)	1000	
Abdominal pain	2 (1.1)	2 (2.6)	0 (0.0)	0.179	
Syncope	1 (0.5)	1 (1.3)	0 (0.0)	0.425	
Rhinitis	1 (0.5)	1 (1.3)	0 (0.0)	0.425	
Muscle pain	1 (0.5)	0 (0.0)	1 (1.3)	1000	

Abbreviations: BTR, breakthrough reaction; DHR, drug hypersensitivity reaction; E, ethambutol; H, Isoniazid; R, Rifampicine; Z, pyrazinamide.

a percentages shown in the table are column based.

b p-values reflect comparisons between: BTR positive vs BTR negative patient groups.

c Independent samples *t*-test.

d Pearson chi-squared test.

e Fisher exact test

### Treatment and initial DHR characteristics

One hundred and seventy-nine patients had a total of 213 DHRs; 148 had 1, 28 had 2, and 3 had 3 reactions. Median total reaction number was 1.^1^, ^2^, ^3^

The severity of the initial DHR was grade 1 in 146 (81.6%), grade 2 in 22 (12.3%), and grade 3 in 11 (6.1%) patients.

The mean interval between antituberculosis therapy initiation and initial DHR was 6,84 ± 12,12 (1–90) days. Initial DHR was on the first day of therapy in 86 (48.04%), within the first week in 132 (73.7%), and within the first four weeks in 171 (95.5%) patients. The interval between the last drug dose and initial DHR was <1 h in 83 (46.4%), 1–6 h in 70 (39.1%), and 6–12 h in 26 (14.5%) patients.

Considering the 26 patients who had a DHR chronologically between 6 and 12 h; 16 patients had urticaria, and 10 patients had pruritus with erythema. Urticaria was accompanied by angioedema in 4 patients, dyspnea in 1 patient, vomiting in 1 patient, and anaphylaxis with hypotension in 1 patient. DHR was on the first day of therapy in 6 (23.1%) and within the first week in 12 (46.2%) of these 26 patients.

During the initial DHR, 91.6% (n = 164) of the patients were on standard combination therapy with HRZE. Non-standard medication combinations were as follows; HRZE with streptomycin 4.5% (n = 8), HR 1.7% (n = 3), HRE, HZE, HES, RZ with moxifloxacine each 0.6% (n = 1). Treatment interruption before DHR was present in 10 (5.6%) patients; all were due to drug toxicity (See Table 2).

### Desensitization outcomes and BTR characteristics

A total of 690 drug desensitizations; 173 H, 173 R, 173 Z, and 171 E desensitizations were performed in 179 patients. Drug provocation tests with streptomycin (n = 9) and moxifloxacine (n = 1) were negative in patients who used these drugs during initial DHR. Desensitizations were completed without any BTR in 103 (57.5%) patients, while 76 (42.5%) patients had 94 BTRs during desensitizations.

Detailed demographics, disease, treatment, and DHR characteristics of patients with and without a BTR are shown in Table 2. No statisticaly significant difference was found in BTR-positive and BTR-negative patients in terms of these variables.

Four patients had known culprit drug before, and desensitization was performed with these drugs, 3 of 4 (75%) patients completed the protocol without any BTR. In 175 (97.8%) patients for whom the culprit drug was unknown, desensitization was performed with all first-line antituberculosis drugs used during the DHR, and 100 of 175 (57.1%) patients completed the protocol without any BTR. There was no significant difference in BTR development in patients with (n = 4) and without (n = 175) known culprit drug before (p = 0.638).

Z was the most common culprit of BTRs (59.6%, 56 of 94). BTR development rates during desensitizations with H, R, Z, and E were 5.2% (9 of 173), 10.4% (18 of 173), 32.4% (56 of 173), and 6.4% (11 of 171) respectively. BTR severities were grade 1 in 79 (84%), grade 2 in 11 (11.7%), and grade 3 in 4 (4.3%) reactions. Most of the BTRs were after the last step of the protocol. BTR characteristics of each drug are shown in Table 3.

**Table 3:** BTR characteristics of each drug^a^.

Variables	Isoniazid	Rifampicin	Pyrazinamide	Ethambutol
Number of desensitizations, n	173	173	173	171
Number of BTRs, n (%)	9 (5.2)	18 (10.4)	56 (32.4)	11 (6.4)
BTR severity n (%) a				
Grade 1	5 (55.6)	14 (77.8)	50 (89.3)	10 (90.9)
Grade 2	3 (33.3)	4 (22.2)	4 (7.1)	0 (0.0)
Grade 3	1 (11.1)	0 (0.0)	2 (3.6)	1 (9.1)
Last step before BTR; n(%) a				
2	0 (0.0)	1 (5.6)	0 (0.0)	0 (0.0)
3	1 (11.1)	2 (11.1)	0 (0.0)	0 (0.0)
4	2 (22.2)	1 (5.6)	3 (5.4)	0 (0.0)
5	2 (22.2)	1 (5.6)	2 (3.6)	1 (9.1)
6	4 (44.4)	13 (72.2)	8 (14.3)	2 (18.2)
7	ND	ND	12 (21.4)	1 (9.1)
8	ND	ND	31 (55.4)	7 (63.6)
Interval between last administered step and BTR, n(%) a				
<30 min	3 (33.3)	6 (33.3)	19 (33.9)	4 (36.4)
30–60 min	2 (22.2)	4 (22.2)	15 (26.8)	2 (18.2)
60–120 min	1 (11.1)	–	8 (14.3)	–
>120 min	3 (33.3)	8 (44.4)	14 (25)	5 (45.5)

Abbreviations: BTR, breakthrough reaction, ND, not defined.

a Percentages shown in the table are column based

Sixteen patients had BTRs with multiple drugs; 1 patient with all 4 drugs and 15 patients with 2 drugs out of which 5 with RZ, 4 with EZ, and each 2 with HZ, RE, and HR. There was no significant difference in terms of demographics, disease, and DHR characteristics between patients who had BTRs with multiple drugs or not (p > 0.05). The frequency of a BTR with a single drug was significantly higher in Z than in other drugs (p < 0.001) (Table 4).

**Table 4:** Single and multiple drug BTR distrubitions of each drug.

Drugs	BTR, total n (%)	BTR, single drug n (%)	BTR, multiple drug n (%)	p
Isoniazid	9 (100)	4 (44.4%)	5 (55.6%)	1000
Rifampicin	18 (100)	8 (44.4%)	10 (55.6%)	0.505
Pyrazinamide	56 (100)	44 (78.6%)	12 (21.4%)	<0.001
Ethambutol	11 (100)	4 (36.4%)	7 (63.6%)	0.201

Abbreviations: BTR, breakthrough reaction

The statistically significant variables in univariate analysis of demographics, disease, and DHR characteristics between patients with and without BTRs according to each drug are shown in Table 5. Patients who had BTRs with R were further analyzed to detemine the independent risk factors of BTRs, and a multivariate regression analysis was performed in the model established with treatment interruption before initial DHR, the interval between treatment initiation and DHR (days), whether standard HRZE combination was used during the initial DHR, concomitant BTR with H or E. Interval between treatment initiation and DHR and concomitant BTR with E were found to have increased the risk for BTR caused by R (p = 0.017 OR = 1.055, 95%CI = 1010–1102 and p = 0.010 OR = 8036 95%CI = 1640–39 373 respectively) (Table 6).

**Table 5:** Statistically significant characteristics in patients with and without BTR during desensitization according to each drug.

ISONIAZID				
Variables	BTR with Isoniazid		p^b^	Statistical method used
	Positive, n (%)^a^	Negative, n (%)^a^		
Previous drug allergy, n (%)			0.046	c
Yes	3 (33.3)	14 (8.5)		
No	6 (66.7)	150 (91.5)		
BTR with Rifampicin, n (%)			0,041	c
Yes	3 (33.3)	13 (8.1)		
No	6 (66.7)	148 (91.9)		
**RIFAMPICIN**				
Variables	BTR with Rifampicin		p^b^	Statistical method used
	Positive, n (%)^a^	Negative, n (%)^a^		
Treatment interruption before initial DHR, n (%)			0.005	c
Yes	4 (22.2)	4 (2.6)		
No	14 (77.8)	151 (97.4)		
Interval between treatment initiation and DHR, median days (min-max)	7 (1–90)	1 (1–50)	0.006	d
Regimen used during DHR, n (%)			0.001	c
Standard HRZE combination	12 (66.7)	148 (95.5)		
Non-standard combination	6 (33.3)	7 (4.5)		
BTR with Isoniazid, n (%)			0.041	c
Yes	3 (18.8)	6 (3.9)		
No	13 (81.3)	148 (96.1)		
BTR with Ethambutol, n (%)			0.042	c
Yes	3 (23.1)	8 (5.2)		
No	10 (76.9)	146 (94.8)		
**PYRAZINAMIDE**				
Variables	BTR with Pyrazinamide		p^b^	Statistical method used
	Positive, n (%)^a^	Negative, n (%)^a^		
Interval between last drug dose and DHR, n (%)			0.027	e
<1 h	33 (58.9)	48 (41.0)		
>1 h	23 (41.1)	69 (59.0)		
**ETHAMBUTOL**				
Variables	BTR with Ethambutol		p^b^	Statistical method used
	Positive, n (%)	Negative, n (%)		
BTR with Rifampicin, n (%)			0,042	c
Yes	3 (27.3)	10 (6.4)		
No	8 (72.7)	146 (93.6)		

Abbreviations: BTR, breakthrough reaction; DHR, drug hypersensitivity reaction.

a Percentages shown in the table are column based.

b p-values reflect comparisons between: BTR positive vs BTR negative patient groups.

c Fischer exact test.

d Mann whitney u test.

e Pearson chi-squared test

**Table 6:** Multivariate regression analysis results of patients who had BTR with Rifampicin.

	Multivariate Analysis	
Variables	OR (CI)	p
Treatment interruption before initial DHR	2247 (0.164–30.809)	0.544
Interval between treatment initiation and DHR, median days	1055 (1010–1102)	0.017
Regimen used during DHR		
Standard HRZE combination	0.177 (0.028–1128)	0.067
Non-standard combination		
BTR with Isoniazid	5391 (0.819–35.476)	0.080
BTR with Ethambutol	8036 (1640–39.373)	0.010
Constant	0.682	0.648

Abbreviations: BTR, breakthrough reaction; CI,Confidence Interval; DHR, drug hypersensitivity reaction; E, ethambutol; H, Isoniazid; OR, Odds Ratio; R, Rifampicine; Z, pyrazinamide

In 40 of 76 patients who had BTRs, the desensitization procedure was not continued due to the primary physician's choice of alternative therapy. Twenty-seven of these patients had BTRs more than 2 h after completion of the procedure. Desensitization was successfully completed after 33 of 42 BTRs in 29 of 36 (80.6%) patients who continued the procedure after a BTR. The desensitization success after a BTR for H, R, Z, and E was 80.0% (4 of 5), 91.7% (11of 12), 71.4% (15 of 21), and 75.0% (3 of 4) respectively. The desensitization procedure was successfully completed in 132 of 139 (95%) patients; 103 patients with no BTR, and 29 of 36 patients who successfully completed the procedure after a BTR.

## Discussion

In this study, we retrospectively reported the characteristics of DHRs, the outcomes of rapid desensitizations, and the characteristics and risk factors for BTRs with first-line antituberculosis drugs in patients with active tuberculosis infection. To the best of our knowledge, this study has the largest patient population desensitized to antituberculosis drugs in the literature.

There was female dominance in the study group with a female-to-male ratio of 2.3:1. We previously reported that female gender is a risk factor for immediate-type DHR with first-line antituberculosis drugs.^23^ Some other studies reported that the female gender was a risk of antituberculosis DHR.^3^^,^^4^^,^^26^

Immediate-type DHRs with antituberculosis drugs were reported to be developed in the early treatment period within days to a few weeks.^15^^,^^26^^,^^27^ Compatible with previous reports, in this study initial DHR was developed within the first week in 132 (73.7%) cases; we also realized that it was on the first day of therapy in 86 (48.04%) cases. A previous study also reported that 14.7% of the antituberculosis DHRs were developed on the first day of therapy.^17^ These results suggest that some of the immediate reactions develop without requiring a sensitization period and may be non-IgE mediated. Besides there are reports of skin test positivity as an evidence of sensitization and IgE-mediated DHR with antituberculosis drugs.^28^, ^29^, ^30^, ^31^ We did not perform skin tests in this study because our aim was not to identify the culprit drug but to provide rapid and effective management of patients and also, as a parenteral drug required for performing intradermal tests only the parenteral form of R is available in our country. Further studies are required to elucidate the mechanisms of immediate-type DHRs with antituberculosis drugs.

Presentation of DHRs may vary. They can be classified based on chronology, mechanism, and clinical phenotypes.^10^ Chronologically, reactions can be classified as immedite and delayed (non-immediate) reactions.^10^ Immediate reactions typically occur within 1 h but, in some cases up to 6 h after the last drug administration and phenotypically present with cutaneous symptoms (eg, flushing, pruritus, urticaria, angioedema), rhinitis, conjunctivitis, respiratory symptoms (dyspnea, bronchospasm), gastrointestinal symptoms (nausea, vomiting, diarrhea, abdominal pain), or anaphylaxis.^10^^,^^11^^,^^32^ Delayed (non-immediate) DHRs may occur at any time from 1 h after the initial drug administration, commonly after many days or, in some cases, weeks of treatment.^10^^,^^11^^,^^32^ The most common clinical presentations are maculopapular exanthemas and delayed urticaria.^11^ Although classification based on chronology and clinical phenotypes is important in clinical practice for workup planning, it may not always reflect the underlying mechanism. Several factors influence the elicitation period of reactions, including unreliable history, the administration route of drug, the role of drug metabolites, and the presence of co-factors or co-prescribed drugs may accelerate or slow down the onset or progression of a reaction.^11^^,^^33^^,^^34^

In our previous study, we demonstrated the effectiveness of our protocols in patients who had immediate type DHR to first-line antituberculosis drugs within 1 h after drug intake. One of our aims in this study was to evaluate the efficacy of this protocols in a larger group of patients phenotypically compatible with immediate-type DHR regardless of chronology. Maculopapular eruption and other forms of delayed-type DHRs were excluded from the study. In this study 26 (14.5%) of the patients had DHR chronologically 6–12 h after last drug intake; 16 of these patients had urticaria, and 10 had pruritus with erythema. There was no difference in terms of BTR development in patients who had a reaction before or after 6 h chronologically (p = 0.156).

There are a limited number of studies focusing on immediate-type DHRs with antituberculosis drugs. In most studies, antituberculosis DHRs were reported as cutaneous adverse drug reactions and most of these are associated with nonimmediate-type DHRs. The most common culprits of cutaneous adverse drug reactions were reported differently in these studies.^4^, ^5^, ^6^, ^7^^,^^15^^,^^35^^,^^36^ Considering the studies in which the majority of the study group consisted of patients compatible with immediate-type DHR, Smadhi et al reported Z as the most common culprit after reintroduction, and Katran et al reported Z as the most common culprit of BTRs during desensitizations.^17^^,^^29^ However in a study reporting adverse drug reactions to first-line antituberculosis drugs in 17 843 cases, R was the most common culprit of common adverse reactions and also DHRs such as rash, pruritus, urticaria, fever, eosinophilia, and dyspnea.^27^ In our study Z was found to be the most common culprit of BTRs with a BTR development rate of 32.4% and had the lowest desensitization success after a BTR (71.4%). However BTRs caused by Z were significantly seen as single drug BTR (p < 0.001).

Tuberculosis therapy requires multiple drug administration concomitantly, and hypersensitivity to multiple drugs may develop simultaneously. Multiple drug hypersensitivity was reported at a substantial level with antituberculosis drugs.^11^^,^^13^, ^14^, ^15^, ^16^, ^17^, ^18^^,^^29^ In our study, BTRs with multiple drugs were detected in 16 of 76 (21.1%) patients, and one patient had BTRs with all first-line antituberculosis drugs. Smadhi et al. reported multiple DHR in 22% of patients after reintroduction, and two of these patients developed DHR with all first-line antituberculosis drugs.^17^

Data about antituberculosis DHR and its management is limited. Although drug provocation testing is the gold standard method to detect and remove the culprit drug from therapy, it carries a risk of reintroduction reactions. Lehloenya et al investigated antituberculosis drug reintroduction in patients who developed cutaneous reactions. Half the patients had reintroduction reactions, and 52% of reactions were moderate to severe. The rate of DHR with antituberculosis drugs may actually be higher in their study because 39 patients were on cotrimoxazole during the initial DHR but not reintroduced in 29 patients.^35^ Yee et al rechallenged antituberculosis drugs in 11 of 21 patients with rash or drug fever only 1 successfully.^4^

In case of a reintroduction reaction, before a new drug provocation, it is necessary to wait for the complete resolution of symptoms and also eliminate the drugs used to treat the reaction. This strategy causes a delay in starting standard therapy, especially in patients with multiple drug hypersensitivity, owing to the time needed for treatment of the repeated reactions. In addition, even if the culprit drug(s) are detected, it is not possible to easily remove them from the treatment. Therefore, the priority in this patient group must be to ensure the reuse of drugs rather than finding the culprit. Aberer and Kranke suggested desensitization rather than detailed allergy testing as the initial method in patients who have cystic fibrosis or tuberculosis in whom the drug concerned is more effective than the alternatives.^21^ The recommendations of Bermingham et al in the management of type-1 DHR with antituberculosis drugs are as follows: i) desensitization with culprit drug and sequencial rechallenge with other drugs if the culprit is known, ii) desensitization with all drugs if the culprit is not known.^22^

There is no standardized instutional rapid desensitization protocol for immediate type DHRs with antituberculosis drugs. There are some protocols implemented in cases or small-sized case series after determination of the culprit drug(s), and most of them consist of more than 10 steps and have longer implementation times than our protocols.^28^^,^^30^^,^^37^, ^38^, ^39^, ^40^ To the best of our knowledge, our protocols are currently the only protocols that have been planned to reuse the antituberculosis drugs without determining the offending drug.

With the protocols used in this study, 57.3% (103 of 179) of the patients successfully completed desensitization without any BTRs. In 36 patients who had BTRs, desensitization was continued after appropriate treatment of the reaction, and 29 of them (80.6%) successfully completed desensitization. The overall success rate of the desensitizations were found to be 95% (132 of 139). The success of our protocols has also been verified with a study conducted in a different center. Katran et al reported that HRZE was started without any problems in 49 of 81 (60.5%) patients with mild immediate reaction.^29^ In addition to be successful, these protocols are also advantageous because of their short implementation time.

Desensitization procedures that start from lower doses, involve more steps, or have shorter intervals between doses may improve safety, but these adaptations will also prolong the implementation time.^41^ Short implementation time is important in these groups of patients. Tuberculosis is a disease with a risk of transmission, so the desensitization procedure of these patients is carried out in an isolated room during daytime working hours in our allergy clinic. At the end of the procedure, the patients are monitored in our clinic for 2 h in case of a possible reaction, and then they are hospitalized in the isolated tuberculosis patient service in our hospital.

In this study, risk factors for BTR development according to each drug were also evaluated, and interval between treatment initiation and DHR and concomitant BTR with E were found to have increased the risk for BTR caused by R. To the best of our knowledge, our study is the only study in the literature that indicates the risk factors for BTR seen during antituberculosis drug desensitization. Further studies are needed to understand the contribution of these statistically significant data to clinical practice.

However, there are several limitations of this study. The first limitation is the retrospective design of the study. The second limitation is in some patients who had BTR during the procedure the primary physician decided to replace the offending drug with an alternative and the protocol was not continued after a BTR. We could not evaluate the overall success of the protocols in these patients.

## Conclusion

In conclusion, data about immediate type DHR due to antituberculosis drugs and their management are limited. Multiple drug hypersensitivity is substantial and one of the major problems in the management of patients. Due to limitations of alternative therapies, it is important to ensure the reuse of first-line drugs. Desensitization with all drugs used during DHR is an important option for the rapid and effective management of patients. We have demonstrated that our rapid desensitization protocols are effective and safe with the advantage of having short implementation time in patients phenotypically compatible with immediate type DHR with first-line antituberculosis drugs regardless of chronologically and may guide rapid and effective management of the patients. In patients for whom our rapid desensitization protocols failed, using different desensitization protocols that start from lower doses, involve more steps, or have shorter intervals between doses may be considered. Another option is switching to second-line therapy.

## Abbreviations

BTR: Breakthrough reaction; DHR: Drug hypersensitivity reaction; E: Ethambutol; H: Isoniazid; R: Rifampicin; S: Streptomycin; Z: Pyrazinamide.

## Funding

The authors have no funding source.

## Data availability statement

Available, if requested.

## Author contributions

Gozde KOYCU BUHARI; conception and design of the study, data generation, analysis and interpretation of the data and preparation and revision of the manuscript.

Ferda ONER ERKEKOL; conception and design of the study, analysis and interpretation of the data and preparation and revision of the manuscript.

Ilkay KOCA KALKAN; data generation, analysis and interpretation of the data and preparation of the manuscript.

Hale ATES; data generation, analysis and interpretation of the data and preparation of the manuscript.

Gurgun Tugce VURAL SOLAK; data generation, analysis and interpretation of the data and preparation of the manuscript.

Ozgur AKKALE; data generation, analysis and interpretation of the data and preparation of the manuscript.

Kurtulus AKSU; data generation, analysis and interpretation of the data and preparation of the manuscript.

## Ethics approval

The study was approved by local ethics committee (approval number 2012-KAEK-15/2614).

## Authors’ consent for publication

All authors have read the manuscript and approved its submission.

All authors gave consent for the publication of the manuscript.

All authors met the requirements for authorship criteria.

The authors confirm the manuscript is original, has not been published before and has not been posted to a preprint server.

The manuscript is not under consideration by any other journal at the same time and it has not been accepted for publication elsewhere.

## Declaration of competing interest

The authors have declared no conflict of interest regarding to this manuscript.
